# Hiding in plain sight: the biomolecular identification of pinniped use in medieval manuscripts

**DOI:** 10.1098/rsos.241090

**Published:** 2025-04-09

**Authors:** Élodie Lévêque, Matthew D. Teasdale, Sarah Fiddyment, Maiken Hemme Bro-Jørgensen, Luke Spindler, Ruairidh Macleod, François Bougard, Morten Tange Olsen, Matthew Collins

**Affiliations:** ^1^ HiCSA, Université Paris 1 Panthéon-Sorbonne, Paris, France; ^2^ Institut de Recherche et d’Histoire des Textes, Paris, Île-de-France, France; ^3^ Evolutionary Genomics, Globe Institute, University of Copenhagen, Copenhagen, Denmark; ^4^ McDonald Institute for Archaeological Research, University of Cambridge, Cambridge, UK; ^5^ Bioinformatics Support Unit, Faculty of Medical Sciences, Newcastle University, Newcastle Upon Tyne, UK; ^6^ BioArCh, Department of Archaeology, University of York, York, UK; ^7^ School of Biochemistry and Cell Biology, University College Cork, Cork, Ireland; ^8^ School of Archaeological and Forensic Sciences, University of Bradford, Bradford, UK

**Keywords:** medieval manuscripts, sealskin, pinniped, Romanesque bindings, Norse trade networks, biocodicology

## Abstract

The survival of medieval manuscripts in their original bindings remains a rare occurrence. Taking advantage of the diversity of bindings in Cistercian libraries such as Clairvaux and its daughter abbeys during the twelfth and thirteenth centuries, this study focuses on the biocodicological analysis of medieval manuscript bindings, with particular emphasis on the use of sealskins. Using innovative methods such as electrostatic zooarchaeology by mass spectrometry (eZooMS) and ancient DNA (aDNA) analysis, this research identifies the animal species and origin of the leather used in these bindings as predominantly pinniped (seal) species. In particular, the collagen-based eZooMS technique facilitated the classification of seven chemises into the pinniped clade, although species identification remained elusive, except in one additional case where a bearded seal (*Erignathus barbatus*) was definitively identified. aDNA analysis was instrumental in verifying the origin of the sealskins, with four samples identified as harbour seals and a single sample as a harp seal and sourced to (contemporary) populations in Scandinavia, Scotland and Iceland or Greenland. This geographical inference supports the notion of a robust medieval trade network that went well beyond local sourcing, linking the Cistercians to wider economic circuits that included fur trade with the Norse. The study, therefore, highlights the use of an unexpected skin (seal) from an unexpected source (the northwestern Atlantic). The widespread use of sealskins in Cistercian libraries such as Clairvaux and its daughter abbeys during the twelfth and thirteenth centuries hints at broader trade networks that brought, for example, walrus ivory from the far north into continental Europe. This integration of the biological sciences into the study of historical manuscripts not only provides a clearer picture of the material culture of medieval Europe, but also illustrates the extensive trade networks that Cistercian monasteries were part of, challenging previous assumptions about local resource use in manuscript production.

## 1. Introduction

Medieval manuscripts provide a direct link to the past, enabling users of today’s reading rooms to handle the same parchment pages as the object’s creators. This tangible connection enlivens the historical study of book production, as the complete set of choices that allowed the creation of a manuscript can still be seen today. Moreover, with an appropriate chronology of objects, researchers can track the development of the technology of book production through time.

In the twelfth and thirteenth centuries, manuscript texts circulated in the form of the codex, which had firmly established itself as the standard medium for writing in the Western world since the fifth century. Unlike earlier tablets, where text was often copied after assembly, codices in the Middle Ages were typically written before the gatherings were bound together. Manuscript pages were made of parchment, derived from animal skins processed without tanning: dehaired, smoothed and stretched under tension to create a durable surface suitable for writing on both sides (opisthographic). Once copied, the manuscript was protected by a binding, a material and structural ensemble that preserved the text as a unified whole and facilitated reading. The materials used—wood, leather, cords and threads—were chosen both for their functional role in the structure and based on their local or commercial availability.

Books serve as both vessels of content and cultural artefacts, preserving historical insights. The codex, the dominant European book form since antiquity, consists of folded sheets bound at the spine with covers, yet its structural details often go unnoticed. Binding, essential to the codex’s function, evolved from simple thread loops to complex sewn structures, adapting to technological and societal shifts.

Interest in bookbinding history began in the nineteenth century, initially focused on decorative aspects. Scholars emphasized aesthetics, and until the 1970s, the field was mostly seen through the lens of decoration. While decorative bindings are still valued, there is a growing recognition of the need for a holistic approach, particularly for early medieval bindings, where decoration is minimal. A new archaeological approach considers the book itself as an artefact, not merely as a repository for text. By examining its form, structure and materials, scholars can uncover valuable cultural insights embedded in its physical construction, treating the book as a historical object with stories beyond its written content. This perspective reveals layers of information about the technologies, craftsmanship and social contexts in which books were produced and used.

Medieval Western European bookbinding techniques, particularly Romanesque bindings that emerged in the eleventh century, signify a pivotal evolution in the craft. The collection of manuscripts from the Library of Clairvaux Abbey, founded in 1115 in the Val d’Absinthe, Champagne, France, offers a rich source to understand these developments. The collection, estimated to have comprised approximately 1000 volumes at the beginning of the fourteenth century, provides a wealth of information for studying the evolution of bookbinding practices during this period. Today, the collection represents a remarkable corpus of 1450 surviving medieval books, with approximately 50% still in their original bindings. Of these, 168 are Romanesque bindings from the twelfth to the fourteenth century, with 28 almost intact. Until the mid-thirteenth century, the majority of the manuscripts were produced at the single scriptorium of Clairvaux. Consequently, this archive enables a comprehensive investigation of the manufacturing techniques employed within a single workshop [1].

**Figure 1.**
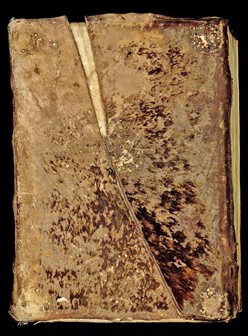
Romanesque binding from Clairvaux covered with a chemise with hairs on (Médiathèque du Grand Troyes, ms. 35, ca. 1141−1200), sample EL53.

The bindings of the Clairvaux Romanesque manuscripts exhibit a distinctive feature: they are protected by a secondary cover, or chemise, made of an atypical leather on which hairs remain (figure 1). In historical library catalogues, the skin is described as being made from either boar or deer. However, upon closer examination, the distribution of the hair follicles on the covers was found to differ from that of either animal.

This ambiguity in identification prompted the adoption of biocodicology—the study of the biological information stored in manuscripts [2]—to facilitate a further examination of the materiality of these objects. Electrostatic zooarchaeology by mass spectrometry (eZooMS) and ancient DNA (aDNA) were applied to, respectively, seven and nine of the skins from Clairvaux (held in Médiathèque du Grand Troyes, France) and its daughter house, Clairmarais (manuscripts held in Bibliothèque de St-Omer, France) (table 1).

**Table 1.** Summary of sample identification and results.

collection and call number	origin of binding’s manufacture (abbey)	date of ms	ID technique	species ID
Montpellier H2	Clairvaux	1140−1275	visual ID (microscope)	pinniped
Montpellier H3 1			visual ID (microscope)	pinniped
Montpellier H3 2			visual ID (microscope)	pinniped
Médiathèque du Grand Troyes, ms 5			eZooMS	pinniped
Médiathèque du Grand Troyes, ms 31			eZooMS, aDNA (EL53)	harbour seal
Médiathèque du Grand Troyes, ms 35			eZooMS, aDNA (EL51)	harbour seal
Médiathèque du Grand Troyes, ms 37			visual ID (microscope)	pinniped
Médiathèque du Grand Troyes, ms 40 (2)			visual ID (microscope)	pinniped
Médiathèque du Grand Troyes, ms 40 (3)			visual ID (microscope)	pinniped
Médiathèque du Grand Troyes, ms 40 (4)			visual ID (microscope)	pinniped
Médiathèque du Grand Troyes, ms 40 (5)			visual ID (microscope)	pinniped
Médiathèque du Grand Troyes, ms 40 (6)			visual ID (microscope)	pinniped
Médiathèque du Grand Troyes, ms 40 (7)			eZooMS, aDNA (EL52)	harbour seal
Médiathèque du Grand Troyes, ms 40 (8)			eZooMS, aDNA (EL55)	harbour seal
Médiathèque du Grand Troyes, ms 43 (1)			visual ID (microscope)	pinniped
Médiathèque du Grand Troyes, ms 43 (2)			visual ID (microscope)	pinniped
Médiathèque du Grand Troyes, ms 43 (3)			eZooMS, aDNA (EL54)	harbour seal
Médiathèque du Grand Troyes, ms 57			visual ID (microscope)	pinniped
Médiathèque du Grand Troyes, ms 1535			visual ID (microscope)	pinniped
Bibliothèque de Saint-Omer, ms 701	Clairmarais	1150−1250	eZooMS, aDNA (EL57)	harbour seal
Bibliothèque de Saint-Omer, ms 37			eZooMS, aDNA (EL58)	harp seal
Bibliothèque de Saint-Omer, ms 81			visual ID (microscope)	pinniped
Bibliothèque de Saint-Omer, ms 206			visual ID (microscope)	pinniped
Bibliothèque de Saint-Omer, ms 216			visual ID (microscope)	pinniped
Bibliothèque de Laon, ms 8bis	Vauclair	1150−1250	visual ID (microscope)	pinniped
Bibliothèque de Laon, ms 166			eZooMS	bearded seal
Bibliothèque de Laon, ms 176			visual ID (microscope)	pinniped
British Library Add ms 63 077	Rievaulx	1151−1200	visual ID (microscope)	pinniped
Corpus Christi College, Oxford, ms 209	Fountains	1180−1200	visual ID (microscope)	pinniped
P. Getty Collection, Beda Venerabilis	Byland	1150−1175	visual ID (microscope)	pinniped
Bibliothèque Royale de Belgique BR II 946 BR II 940	Cambron	1155−1200	visual ID (microscope)	pinniped
Bibliothèque publique de Bruges, mss 10, 22, 27, 29, 102, 113, 120, 145, 193, 223, 224	Dunes or Ter Doest	12th C.	visual ID (microscope)	pinniped

Visual microscopic analysis was carried out on a further 13 bindings from Clairvaux (held in Médiathèque du Grand Troyes and Bibliothèque InterUniversitaire de Montpellier, France). The results of these analyses have identified the animal origin of the Romanesque chemises and provided further context to the production of this remarkable collection of manuscripts.

Having identified this specific type of binding, characterized by a furry skin chemise, further research was conducted in other libraries to contextualize our discovery and understand the cultural and historical significance of this material. An additional 23 bindings with similar-looking skins were found in other medieval collections at St Omer and Laon in France, as well as libraries in England and Belgium. While additional examples of ‘hairy’ books were discovered in Scandinavian countries and Ireland, these specimens did not conform to the Romanesque construction style and fell outside the designated time period, leading to their exclusion from this study. These findings were made following DNA analysis of the initial seven examples, which confirmed the species of animal used in each case. DNA analysis was not conducted on subsequent examples due to logistical constraints and ethical considerations related to the preservation of historical artefacts.

## 2. Results

### 2.1. Proteomic analysis: electrostatic zooarchaeology by mass spectrometry

Due to the inability to identify the animal species under a microscope at the start, a micro-invasive biomolecular analysis technique called eZooMS, protein analysis through mass spectrometry, was undertaken for seven Romanesque chemises (Troyes, mss 5, 31, 35, 40 (t.7), 40 (t. 8), 43 (t. 3)). Samples from the flesh side of the skins were collected using polyvinyl chloride (PVC) erasers, from which collagen molecules were extracted [3]. The samples were then analysed by matrix-assisted laser desorption/ionization time-of-flight (MALDI-TOF) mass spectrometry. The collagen sequences were insufficient to allow for identification of species, but did assign all chemises to the suborder Pinnipedia. An additional comparison sample taken from manuscript 166 in Bibliothèque de Laon (Vauclair) was, however, able to confirm the use of a bearded seal skin (*Erignathus barbatus*).

### 2.2. Ancient DNA analysis

The second stage of the biocodicological analysis was an aDNA study with the aim of identifying the species of origin of the Romanesque chemises, using the eZooMS identification of *Pinnipedia* as a starting point. In this analysis, eraser crumbs samples from the seven chemises were subjected to DNA extraction and high-throughput sequencing. The sequencing reads recovered were then compared via FastQ Screen [4] to a range of whole genome and mitochondrial DNA (mtDNA) reference sequences. This analysis confirmed the eZooMS identification of *Pinnipedia* and identified an origin of harbour seal skin (*Phoca vitulina*) for four of the samples and harp seal (*Pagophilus groenlandicus*) for a single chemise. Unfortunately, the remaining two samples provided insufficient data to allow for a confident species identification.

**Figure 2.**
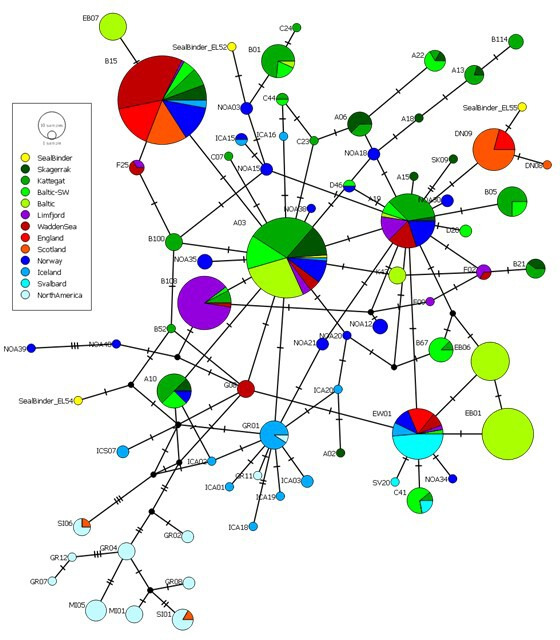
The likely geographical origin of harbour seal skins covering the bindings (EL52, EL53, EL54 and EL55; marked in yellow) was inferred by placing into a median-joining haplotype network with a large sample of mtDNA haplotypes from contemporary North Atlantic harbour seals. Black dots denote missing haplotypes and marks on branches are mutations.

Endogenous DNA was recovered from five of the samples (four harbour; one harp) at sufficient depths to allow for the production of mtDNA consensus sequences (table 2). The likely geographical origin of the four harbour seal binders (EL52, EL53, EL54 and EL55) were inferred by placing them in a median-joining haplotype network with a reference set of mtDNA control region sequences from 495 extant North Atlantic harbour seals (figure 2).

**Figure 3.**
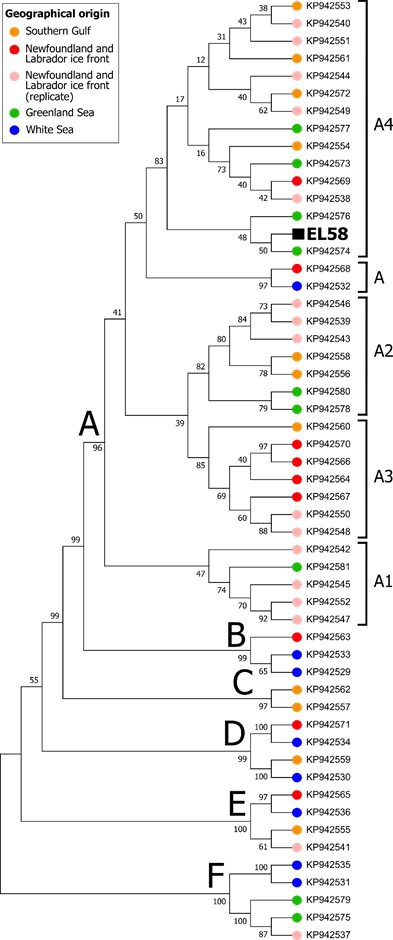
Phylogeny of EL58 together with 53 mitogenomes from modern harp seal populations [7]. GenBank accession numbers are given at each node. The sample nodes are coloured based on their geographical origin [7].

The EL52 sample differs by a single mutation from a contemporary animal (NOA03) taken from Troms county, northern Norway, and two mutations from a relatively abundant mtDNA haplotype found in Kattegat (*n* = 11) and in the Baltic Sea (*n* = 4), suggesting a Scandinavian origin for this skin. Sample EL53 is identical to a highly abundant mtDNA haplotype (*n* = 87, or 17.6% of all animals) in contemporary harbour seals found in the Danish Wadden Sea (*n* = 7), Skagerrak (*n* = 11), Kattegat (*n* = 24), Baltic Sea (*n* = 36), northern Norway (*n* = 8) and a single individual from Iceland (*n* = 1), suggesting a Scandinavian origin. Sample EL54 differs by two mutations from a contemporary animal (B52) collected in Kattegat (Denmark) and by three mutations from an mtDNA haplotype found in the Wadden Sea and another haplotype found in Iceland, suggesting a Nordic origin. EL55 differs by only one mutation from an abundant mtDNA haplotype (*n* = 24, 4.8% of all animals) found in contemporary harbour seal samples originating from Scotland and northern England, suggesting a northern UK origin, most likely Scottish.

Sample EL58 confidently aligned to the harp seal mitochondrial genome with 99.9% coverage (table 2). The phylogeny (figure 3) reveals that EL58 belongs to the widespread A4 mitochondrial clade [7]. However, EL58 falls specifically into a subclade with just two other harp seal samples, which come from the Greenland Sea (KP942574 and KP942576), suggesting Greenland or Iceland as a very likely origin for this skin.

**Table 2.** mtDNA alignment statistics for each sample using an appropriate reference mtDNA. Samples EL1 and EL57 were excluded from the analysis as insufficient data was recovered for a population genetic analysis.

sample	document reference number	species ID	raw reads	mtDNA aligned reads (%)	mtDNA aligned reads PCR duplicates removed (%)	mean depth of coverage mtDNA genome	breadth of coverage of mtDNA genome (≥1X)	possible geographic origin of the animal (mtDNA haplotypes)
EL52	Troyes, Ms 40 [5]	harbour seal	34 386 304	3855 (0.01)	3275 (0.01)	8.8X	99.3%	Scandinavia
EL53	Troyes, Ms 31	harbour seal	42 469 800	40 491 (0.10)	17 319 (0.04)	51.5X	99.9%	Scandinavia
EL54	Troyes, Ms 43 [3]	harbour seal	37 429 038	14 524 (0.04)	9363 (0.03)	25.1X	99.2%	Denmark
EL55	Troyes, Ms 40 [6]	harbour seal	49 666 291	29 844 (0.06)	14 714 (0.03)	44.1X	100%	Scotland
EL58	St Omer, Ms 37	harp seal	31 944 439	4093 (0.01)	3175 (0.01)	8.7X	99.9%	Greenland or Iceland

### 2.3. Metagenomic analysis

Metagenomic classification was undertaken using CCMetagen [8] to further profile shotgun genetic data obtained from these bindings. One of the most consistently prevalent taxa identified across samples was the fungus *Aspergillus*, a ubiquitous environmentally distributed filamentous fungus, known to be also associated with mildew. In particular, *Aspergillus glaucus* appears predominant in samples EL53, EL54 and EL55; this species is the most osmotolerant of its genus [5], which may be linked to the extreme environments associated with the production of bindings. Similarly, a number of samples also showed the presence of *Wallemia ichthyophaga* (particularly EL53 where 45% of all aligned reads were identified to this species) (electronic supplementary material, figure S1), possibly the most halophilic fungus recorded [6]. Potentially linked to the presence of filamentous moulds like *Aspergillus* is the recovery of reads from *Trogium*, commonly known as the paperlouse or booklouse, in EL55, which feeds on these. Additionally, reads were identified in three samples (EL52, EL53 and EL54) mapping to two species of the genus *Liposcelus*, which are also commonly referred to as booklice and an archival pest [9]. Reads from the genus *Anthrenus* (carpet beetles) were also identified (*An. fuscus* in EL53 and EL57, and *An. verbasci* in EL55); carpet beetles are also known for feeding on leather bookbindings [10,11]. Additionally, reads were recovered that mapped to the tephritid fruit fly *Rhagoletis* in a number of samples, and also dust mites (*Pyroglyphidae*).

### 2.4. Examination by visual forensic analysis

Additional species identification of all the remaining covers which had retained their hair could be performed by visual forensic examination using a microscope: a total of 43 bindings were examined, 18 made in Clairvaux, five made in Clairmarais, three in Vauclair, 11 in Dunes or Ter Doest, two in Cambron, one in Byland, one in Fountains and one in Rievaulx. The biocodicological results enabled the comparison of bindings against a reference set of skin samples, and thus identification of the animal species in further samples could be achieved by visual examination without the need for proteomic analysis [12].

**Figure 4.**
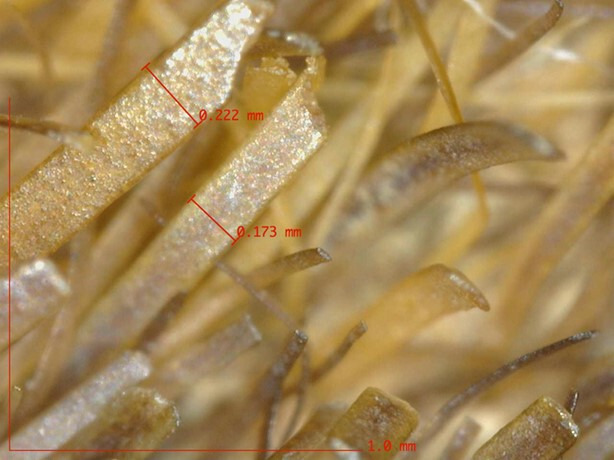
A primary hair, flat at the base. Ms 31 (Médiathèque du Grand Troyes)

**Figure 5.**
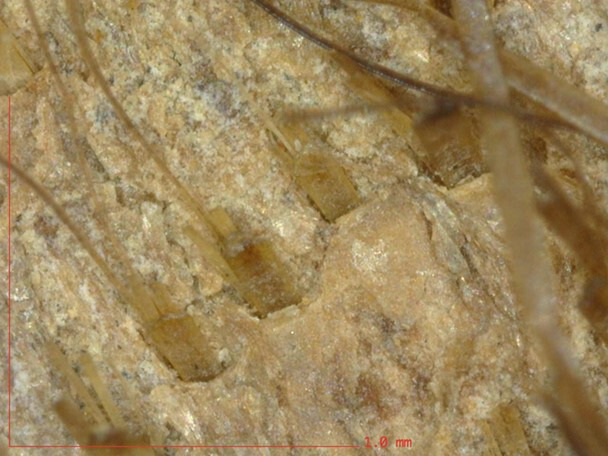
A primary hair with secondary hairs that appear to be lodged in the same follicle exit hole. Ms 31 (Médiathèque du Grand Troyes)

The state of degradation of the hair hindered species identification by microscopic visual examination alone, particularly as the hair length was often not measurable due to degradation (breakage) and/or undulations. Therefore, an *in situ* examination—without sampling—was conducted and revealed a similar structure on all the skins. The surface of the skin, the follicle patterns and the hair fleece, examined under magnification (×20 and ×250), present certain particularities allowing a more reliable identification. The following elements were systematically observed on all skins: the morphology of the hair, its arrangement, its distribution and its density on the surface of the skin; pores and follicles; the structure of the skin (its different layers). It is important to note that some hairs may have disappeared over time (figures 4 and 5).

The observation of the particular characteristics presented by the morphology of the hairs and their implantation, as well as the fat layer at the surface of the skin made it possible to identify, on all the skins examined, the presence of phocids on the bindings made in Clairvaux, Clairmarais and Vauclair [13].

## 3. Discussion

Only a few leather chemises with hairs have survived on the bindings made in Clairvaux. However, the presence of remnants of skin, with hairs, under clasps and traces of sewing, imprinted in the underlying leather of the primary cover on a large number of bindings in the collection, suggests that the manuscripts covered with a sealskin chemise were much more numerous in the twelfth and thirteenth centuries than one might imagine. Originally, 105 Romanesque bindings were covered with a chemise, which represented 63% of the collection at the time. This number is considerable in view of the number of bound manuscripts in Clairvaux at the beginning of the thirteenth century (about 350) [14]. Eighty three percent of these chemises have disappeared with only traces remaining.

The availability of published and unpublished genetic data from modern harbour seal and modern and ancient harp seal populations allowed for geographical provenancing of the animals used to make six of the sealskin chemises to Scandinavia and Scotland (harbour seal) and Iceland or Greenland (harp seal) [15,16]. The assignment of the harbour seal chemise to geographical location is based on the premise that the distribution and connectivity of contemporary harbour seal populations reflect those of the past. Harbour seals exhibit a very high degree of site-fidelity and show marked genetic differences between the North Sea and the Skagerrak–Kattegat–Baltic region [17,18], indicating that the risk of misassignment is low. However, in the absence of ancient data, we cannot rule out that changes in distribution and connectivity have occurred since the Middle Ages. The assignment of the harp seal chemise to geographical origin is less certain, as harp seals are highly migratory, moving thousands of kilometres between foraging and breeding sites and could have been hunted at any point during these migrations. However, our modern and ancient data indicate a Greenlandic, Icelandic or perhaps Barents–White Sea origin. Finally, the sample taken from manuscript 166 in Bibliothèque de Laon (Vauclair) and analysed by eZooMS revealed the presence of the bearded seal (*Erignathus barbatus*), which is a strictly Arctic species. The specimen might have been sourced in Greenland, northern Iceland or the Barents–White Sea region [19], or could alternatively be a vagrant animal, although these are rare in Europe. Bearded seals are rarely recovered in the European zooarchaeological record [20], but often used by prehistoric and contemporary Inuit for materials and consumption (e.g. [21]).

That the skins were drawn from such a wide geographical range outside of Champagne attests to an important trade in sealskins [22]. According to Robert Delort, the sourcing of sealskin on the European market is Swedish or Finnish, from Gotland, Åbo or Viborg, and Norwegian, from Bergen [23]. There is no written record of the purchase or any use of sealskin at Clairvaux Abbey. In order to try to understand the reasons for the presence of such material in Clairvaux, it seemed judicious to us, in the absence of witness texts, to seek its geographical origin of the animal skins through biocodicology.

The skins were found on manuscripts that were produced in areas that are situated on thirteenth-century European trading routes. Clairvaux is located near a land trading route, and was connected to the Champagne fairs, to which Clairvaux was close (and we know that the monks of Clairvaux were allowed to attend). Given the extensive network of the Hanseatic League, which linked various European trading routes, the presence of sealskins in Dunes Abbey and Clairmarais, can be explained, for example.

**Figure 6.**
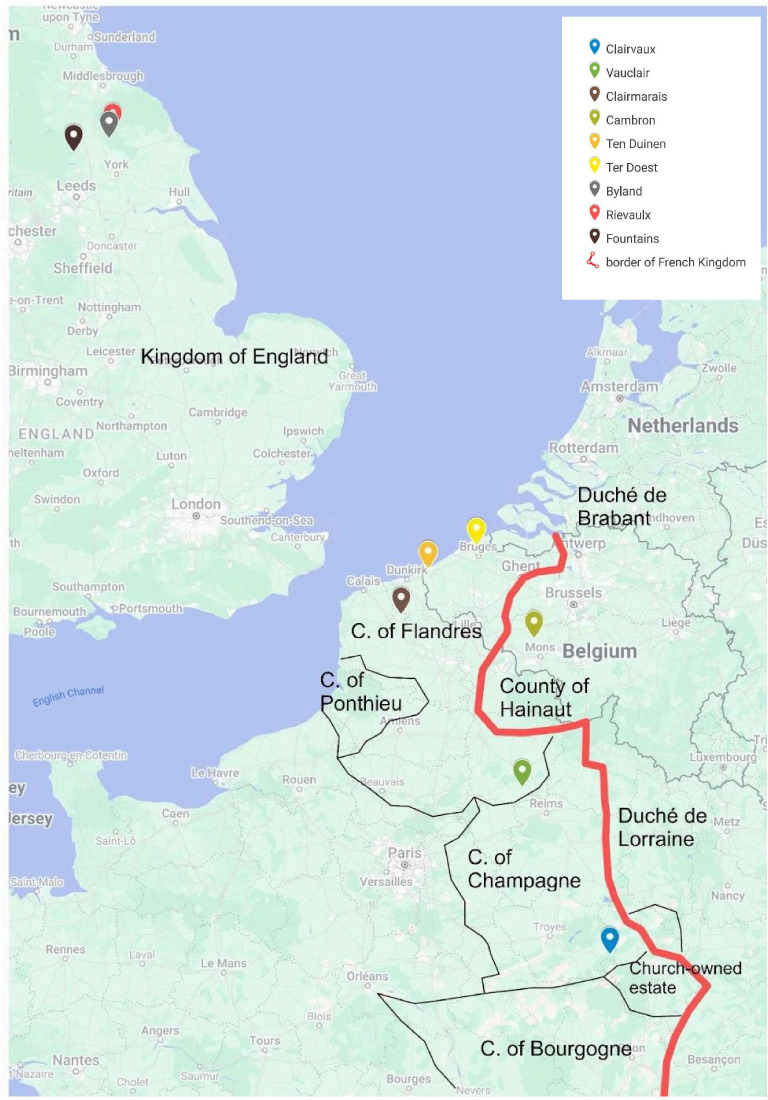
Map showing the places of manufacture of the Romanesque bindings covered with seal skins still kept in library collections. Cîteaux, founded in 1098, the mother house for the Cistercian Order, is only indicated as a reference point. No hairy bindings were found in its manuscripts collection.

**Figure 7.**
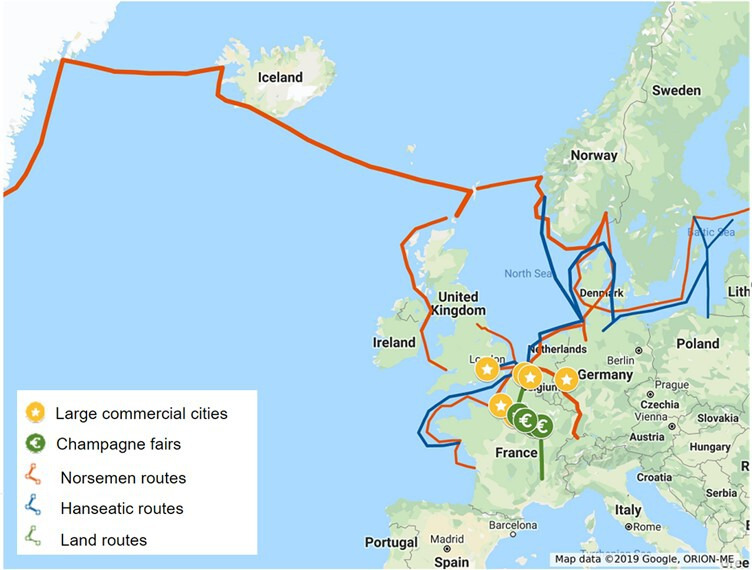
Map showing the medieval trading routes, from Pounds [24] and Bautier [25].

All the samples are additionally located on Norse trading routes (figures 6 and 7). The Norse arrived in Greenland in the ninth century, remaining until the thirteenth century. They traded walrus ivory and fur [16]. Thus, in addition to the commercial exchanges organized by Norse keen on maintaining a trade monopoly, the transport of ivory, skin ropes and seal skins from Greenland to Europe also took place in the form of gifts and tithes. No skins have been recovered from archaeological sites, but some texts mention that sealskins were used to pay tithes to the church [26,27]. Following the Council of Lyon in 1274, an exceptional tithe on all ecclesiastical revenues was levied by Gregory X for a period of six years across Christendom, including in Greenland. The tithe collector (a delegate of Jon of Nidaros) returned four years later ‘with a substantial amount of seal and ox skins, cetacean teeth, whale baleen and leather ropes’: due to a shortage of currency, the contributions were paid in goods. Consulted by the Archbishop of Trondheim, to whom the collector was accountable, Martin IV replied on 4 March 1282, authorizing him to convert these goods into cash (Registers of Martin IV, no. 119). It was during this period that the use of seal skins on the bindings at Clairvaux and its daughter houses ceased [28]. When the climate changed and their hunting techniques could not adapt to the colder conditions during the Little Ice Age, Norse settlements disappeared from Greenland [29]. The cessation of activity may also be reflected in manuscript production. The use of sealskins in bindings was pre-eminent around 1200, but ended just before 1300. Harbour seal populations are non-migratory, and local populations are thus easily depleted if subject to long-term overexploitation (e.g. [30,31]) Additionally, Norse hunting methods in the Arctic were not well suited to the increased sea ice resulting from climate change, making seal populations either drastically reduced or entirely inaccessible to Norse hunters. In contrast, the Thule and Dorset cultures possessed ice-adapted hunting techniques that were more effective under these conditions than those used by the Norse [19]. The presence of a bearded seal among the sampled skins thus reinforces the Norse sourcing of these materials, supporting the theory of their integration into medieval European manuscripts through these extensive Norse trading networks.

Until now, archaeology has mainly focused on the study of seal bones or ivory, rarely examining skin or leather (but see [32]). Our findings align with a recent study on walrus ivories discovered at various Norse-period trade centres across Western Europe, including Trondheim, Bergen, Oslo, Dublin, London, Schleswig and Sigtuna. Although walruses are found across the Arctic, DNA analyses show that ivory from this period originates exclusively from the Arctic North Atlantic, which reached Europe via the Norse in Greenland and Iceland, who may have obtained ivory through both direct hunting and trade with indigenous Arctic populations of North America [15,16,33,34].

**Figure 8.**
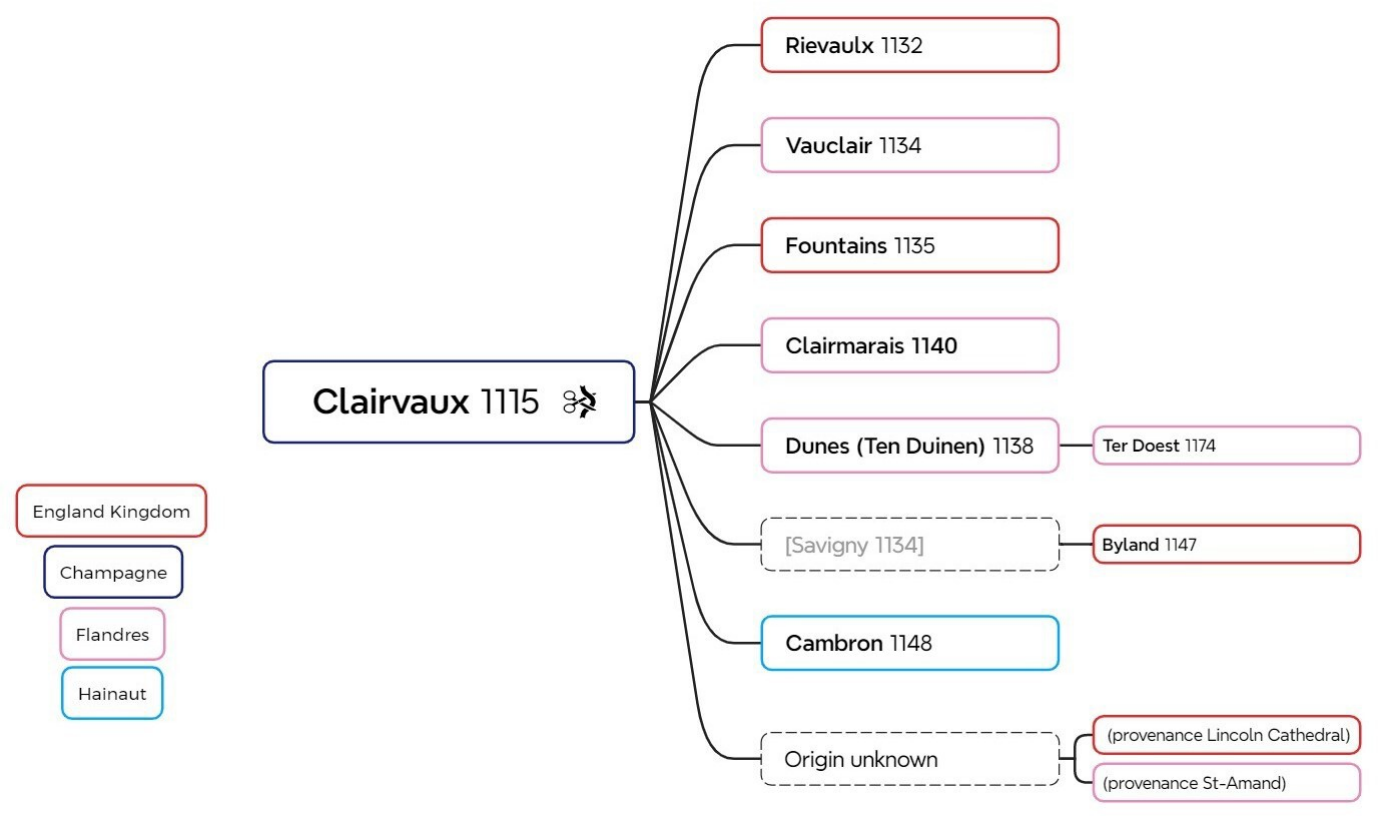
Clairvaux and its daughter abbeys where Romanesque bindings were covered with sealskin.

In the Middle Ages, seal exploitation was common in northern Europe and persists in all Arctic cultures today. As an important source of raw materials and food, these mammals were hunted for their meat, which was consumed, their blubber for heating/fuel and their skin, which was used to make clothing such as anoraks, boots, headgear and gloves. It is not surprising, then, that in the Middle Ages in northern countries, this skin was also used to protect books. Indeed, the use of seal skin for manuscript covers was a common practice in Scandinavian countries and Ireland, where it continued throughout history [22]. The presence of seals in monastic circles is attested in particular by a text by Adomnan of Iona written around 700 AD. He informs us that the seals inhabiting the region close to the northwest Scottish monastery of Iona (Adomnan of Iona, Vita Columbae Abbatis, livre 1, ch. 41) were considered the property of the monks [35]. However, Champagne (or even Vauclair in the Aisne) is an inland region and the use of sealskin is therefore surprising; the lack of archaeological evidence of seal populations on the French coasts in the Middle Ages renders the phenomenon even more unexpected [36]. While the twelfth- and thirteenth-century sealskin bindings are not only attributed to Clairvaux, all of the other libraries (Clairmarais, Vauclair, Bylland, Fountains, Rievaulx, Cambron, Ter Doest and Dunes) (table 1) where they could be found are known to be Cistercian daughter houses of Clairvaux, suggesting a strong connection between the use of these unique bindings and the specific traditions and practices of this particular Clairvaux lineage (figure 8). In Cistercian tradition, a daughter house is a monastery founded by an existing abbey (the mother house), through which it remains spiritually linked and overseen. Abbots from both gather in General Chapters to uphold Cistercian unity and rules. This network of mother and daughter houses enabled the rapid spread of Cistercian communities across Europe in the twelfth and thirteenth centuries, ensuring consistent adherence to the Rule of Saint Benedict and Cistercian ideals. This practice could have been established in Clairvaux as well as imported from England and could be explained by the close relations of the Clairvaux monks with their Irish and English counterparts, and by possible donations of materials, as well as by the circulation of Cistercian ideas, practices and techniques.

**Figure 9.**
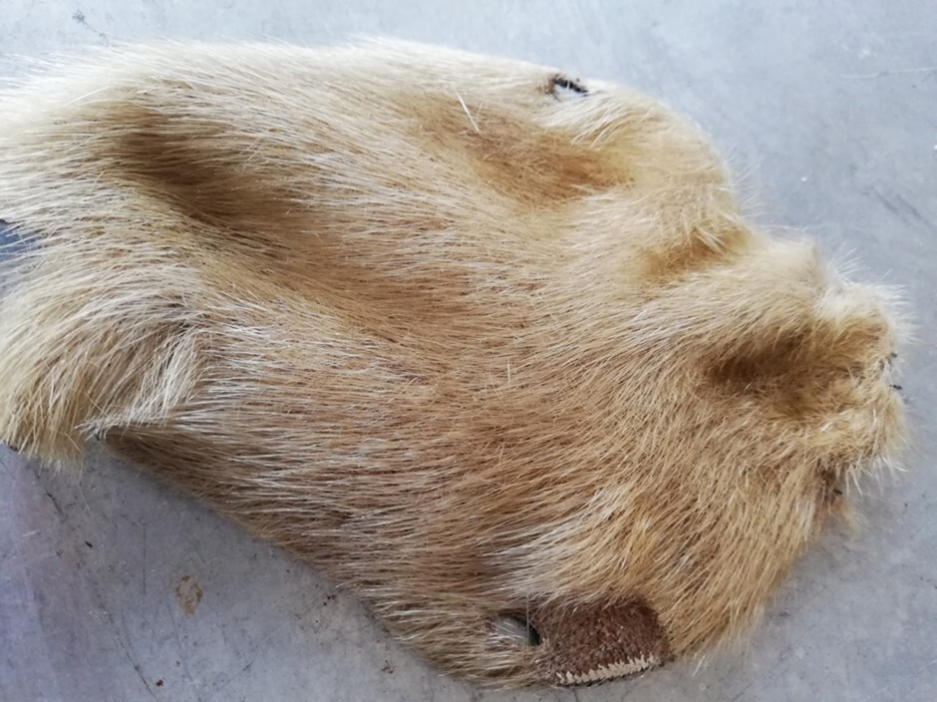
Macroscopic examination of a contemporary sample shows the light colour of the natural hair (this sample has been treated with artisanal, greasy techniques and is undyed). A few millimetres have been shaved from the lower end, exposing the dark appearance of the skin surface under the fur.

**Figure 10.**
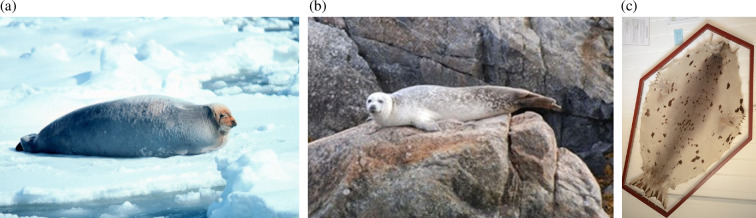
Bearded seal (Wikicommons (a); harbour seal in Greenland, © Morten Tange Olsen (b); young harp seal skin, © Morten Tange Olsen (c)).

**Figure 11.**
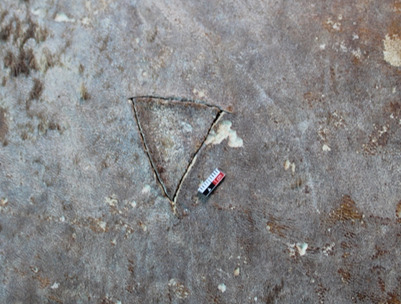
Ms 40 (6), Médiathèque du Grand Troyes, detail of skin patching.

The skins today appear brown in colour, but it is highly unlikely that Cistercians would have covered their books with brown skins: brown was characteristic of the Benedictine order. Cistercians are known for their affinity with white clothing and objects and, therefore, it is likely that pinniped skins were chosen for the chemises due to their light grey or white coloration. The brown coloration observed today is probably a result of degradation over time. The hair has become brittle and fragmented, revealing the underlying layer of subcutaneous fat characteristic of a sealskin. This fatty layer, which appears brown under the light-coloured hair (figure 9), is now more visible due to the hair loss (figures 1 and 5). The biocodicological study of these rare bindings has therefore not only furthered the understanding of the extent of trading networks within which the Cistercians monasteries operated, but also postulated an original physical appearance of the manuscripts. In the Middle Ages, colour density held more significance than hue itself. Light brown or pale grey skins might have been perceived as ‘white’ by monks, who typically did not wear true white. Cistercian monks, for instance, who were meant to dress in white, often wore shades closer to grey, off-white or beige. Similarly, black was rarely a pure black; it was more often a dark blue, grey or brown. The concept of ‘white’ in this period was understood more as a lack of colour, rather than the bright, pure white we think of today [37]. Harbour seals and harp seals display a wide range of light shades, as seen in figure 10. In examining the Clairvaux collection, one notices that sections of leather on covers have been cut and replaced with new patches, creating a sort of patchwork effect (figure 11). On bindings where these outer coverings have vanished, the stitch marks are still visible on the primary layer. While most hair has faded away, the possibility of discoloration around the patched areas remains speculative. However, it seems likely that the patches served to join lighter skin fragments and discard darker ones. The minimal presence of such patches suggests the use of pale, nearly unblemished seal pup skins, which were often favoured for their light tones. Seal skins from the Norse, available through trade or as tithe to the church, offered the desired coloration that set Cistercians apart from orders like the Benedictines, who favoured darker hues.

Contrary to the prevailing assumption that books were crafted from locally sourced materials, it appears that the Cistercians were deeply embedded in a global trading network, acquiring skins through extensive trade exchanges. This observation extends beyond just the bindings to include most of the materials used for both the covers and the parchment of the text blocks. While librarians, palaeographers and codicologists have struggled to identify the origins and provenance of these skins solely through conventional literary sources, the disciplines of biology and biocodicology offer invaluable insight. The integrated approach used in this study not only enriches our knowledge of Cistercian manuscript production but also highlights the broader economic and cultural exchanges of the medieval period.

We have no written records to explain why monks chose to cover these manuscripts in sealskin, nor can we determine if these covers were a sign of particular value or symbolism. All manuscripts produced at the abbey seem to have been bound uniformly, regardless of their content or intrinsic worth and no written explanation for this practice survives. Our understanding must, therefore, rely on what the physical evidence tells us. There is also the question of whether the Cistercian monks—and indeed, medieval French and Western European populations as a whole—knew that the fur they obtained on the market was from seals. Michel Pastoureau (personal conversation, 3 June 2018) speculates that the monks may not have associated the skins covering their manuscripts with the animal described in medieval bestiaries as the ‘sea calf’. The seal was rarely depicted in medieval art and did not hold a place in heraldry, which developed at that time. This lack of representation might have contributed to a detachment from the animal’s true identity, making it difficult for contemporaries to recognize sealskin as a specific material.

## 4. Methods

### 4.1. Samples

An initial focus was placed on manuscripts bound with leather with hairs on, all dated from 1135 to 1250, 19 from the collection of Clairvaux manuscripts, and three from other Cistercian collections, Clairmarais and Vauclair, enhancing the understanding of material composition and historical provenance across these collections.

Sampling was conducted on the flesh side of the leather to avoid contamination from keratin, ensuring only collagen fibres were collected. A representative set of nine manuscripts was selected for eZooMS analysis (including six from Clairvaux), increasing the accuracy of visual identification techniques for the broader collection. Subsequently, seven of these samples were sequenced to provide a comparative analysis (table 2).

### 4.2. Protein analysis

Samples were collected using PVC erasers, following the procedure outlined in Fiddyment *et al.* [3]. Using the established eZooMS technique, samples were first processed with MALDI-TOF MS. The choice of eZooMS is justified by the heritage nature of the collection studied, which requires the use of non-destructive (or micro-invasive) analysis techniques. It also has the advantage of not requiring transportation of the manuscripts from their current storage.

### 4.3. DNA analysis

#### 4.3.1. Extraction

DNA was extracted from eraser crumb samples taken from each document following the protocol of Teasdale *et al.* [38], in a dedicated ancient DNA laboratory at BioArCh, University of York. Illumina sequencing libraries were prepared for each sample following the protocol of Meyer and Kircher [39] as modified by Gamba *et al.* [40]. Libraries were then sequenced on one lane of a HiSeq4000 at the University of Copenhagen.

#### 4.3.2. Sequencing and bioinformatics

FastQ files for each sample were first quality checked using FastQC (https://www.bioinformatics.babraham.ac.uk/projects/fastqc/) [41] and trimmed for adapter sequences using cutadapt [42]. Sequences were first aligned to multiple mtDNA reference genomes using FastQ Screen [4] and then to the predicted source species mtDNA (harbour seal NC_001325, harp seal NC_008429) using BWA (utilizing recommended settings for aDNA [41,43]. Alignments were then filtered for polymerase chain reaction (PCR) duplicates using SAMtools [44], quality controlled using Qualimap 2 [45] and visualized in integrative genomics viewer (IGV) [46]. Final fasta consensus sequences for each sample were produced using angsd [47] and visualized using SeaView [48].

#### 4.3.3. Haplotype network and phylogenetic analysis

The likely geographical origin of the four harbour seal binders was inferred through the construction of a median-joining haplotype network using PopART [49] and a reference dataset of 954 mtDNA haplotypes (422 bp) from contemporary North Atlantic harbour seals compiled from previous studies [50,51] and unpublished data (Olsen MT). The phylogenetic analysis on sample EL58, identified as harp seal, was carried out in MEGA7 [52]. EL58 was aligned with 53 contemporary North Atlantic harp seal mitochondrial genomes [7]. In MEGA, the alignment was conducted using MUSCLE [53], and a maximum likelihood phylogeny was constructed based on the Hasegawa–Kishino–Yano model [54] with 1000 bootstrap repetitions.

#### 4.3.4. Metagenomic profiling

Metagenomic profiling was undertaken on adapter-trimmed and de-duplicated reads by k-mer alignment, using the ConClave sorting method to resolve multi-mapping reads from KMA [55] following the approach in CCMetagen [8]. This used the NCBI *nt* database as a reference dataset, without unclassified environmental sequences. CCMetagen was found to produce a significantly reduced rate of false positives in comparison with other k-mer-based classification methods [56]. KMA was run with the following options in effect: ‘kma-1 t1-mem_mode-and-apm f’. The results were visualized using Krona [57].

## Data Availability

Sequencing data (FASTQ) with host reads removed, MALDI, mtDNA datasets and krona plots of metagenomic data are submitted in Dryad [58] and Zenodo [59]. Supplementary material is available online [60].
